# Supplementary material for: Longitudinal omics data and preclinical treatment suggest the proteasome inhibitor carfilzomib as therapy for ibrutinib-resistant CLL
Source: Nat Commun. 2025 Jan 26;16:1041. doi: 10.1038/s41467-025-56318-7 (PMC11762753; doi:10.1038/s41467-025-56318-7)
Supplement: Supplementary file 1 — Supplementary Information [file 41467_2025_56318_MOESM1_ESM.pdf]

Supplemental Methods

In vivo treatment

Two to three weeks after tumor cell transplantation, tumor load in blood (defined as the number of CD5+CD19+ CLL cells/L) was measured, and mice were assigned to different treatment arms to achieve comparable tumor load in all groups at baseline prior to treatment. Ibrutinib (provided by Pharmacyclics LLC, an AbbVie Company) was administered in drinking water containing sterile control vehicle (1% HP--CD) at a concentration of 0.16 mg/mL, as previously described[1]. Carfilzomib (Selleck Chem) was solubilized in 10% Captisol (sulfobutylether- b-cyclodextrin). Mice were treated with intravenous injections of vehicle control (10% captisol) or carfilzomib (5 mg/kg/d) twice a week.

Collection of tissue samples and preparation of cell suspensions

Withdrawal of peripheral blood from animals during the course of experiment, was drawn weekly or every second week via puncture of the submandibular vein in EDTA-coated tubes and further used for surface staining. For collection of tissue samples at the end of experiments, mice were euthanized by increasing concentrations of carbon dioxide (CO_2). Peripheral blood (PB) was drawn from the submandibular vein or via cardiac puncture and collected in ethylenediaminetetraacetic acid (EDTA)-coated tubes (Sarstedt, Nümbrecht, Germany). Single-cell suspensions from spleens, bone marrow (BM) and inguinal LNs were prepared as previously described[2,3]. BM cells were flushed from femurs with 5 mL of phosphate-buffered saline (PBS)/5% fetal calf serum (FCS). Spleen single-cell suspensions were generated by using the gentleMACS tissue dissociator with Gentle MACS tubes C (Miltenyi Biotec). Single cell suspensions from LNs were prepared by grinding the tissue through 70 µm cell strainers (BD Biosciences, Heidelberg, Germany). Erythrocytes were lysed by using Red blood cell lysis buffer (Mitenyi Biotec).

Flow cytometry

After preparations of single-cell suspensions, cells were incubated with recommended dilutions of antibodies against cell surface proteins in PBS containing 0.1% fixable viability dye (eBioscience, Frankfurt am Main, Germany) for 30 min at 4°C. Cells were fixed using IC fixation buffer (eBioscience), washed and stored at 4°C in the dark until analyzed by flow cytometry. Tumor load, defined as percentage of CD5+CD19+ cells of total CD45+ hematopoietic cells as acquired by flow cytometry, in spleen (SP), lymph nodes (LN), and bone marrow (BM), was compared between control and treated mice.

For labeling of cells in whole blood, 25-50 µl of PB were stained with antibodies specific for surface molecules for 30 min at 4°C, followed by incubation for 10 min with 2 mL of 1x BD FACS lysing solution (BD Biosciences) to remove erythrocytes. After centrifugation, supernatants were carefully aspirated, and pelleted cells were resuspended in 150 µl of 1x BD FACSLysing solution. 50 µl of 123count eBeads (eBioscience) were directly added before data acquisition. Absolute cell numbers in blood were calculated according to the formula: absolute count (cells/µL) = (cell count **x** bead volume x bead concentration)/ (bead Count **x** cell Volume).

For all flow cytometric measurement, data acquisition was done using BD LSRII or BD FACSCanto flow cytometer (BD Biosciences). Median Fluorescence Intensity (MFI) was recorded and normalized by subtracting the MFI of the respective fluorescence-minus-one (FMO) control. Data analysis was performed using FlowJo X 10.0.7 software (FlowJo, Ashland, OR, USA).

For cell sorting, splenocytes were stained for CD45, DCD5 and CD19 surface proteins. CLL cells were defined as $DAPI^-$ $CD45^+$ $CD5^+CD19^+$ and were sorted in PBS with 5% FCS using BD FACSAria II (BD Biosciences) running with FACSDiva Software (BD Biosciences) using a 85 µm nozzle. Purity of sorted cells was typically above 95%.

Negative selection of murine B cells

For magnetic cell sorting, splenocytes were enriched for B cells using EasySep Mouse PanB Cell Isolation Kit (Stem Cell Technologies). Sorted cells were stained for CD45, CD5 and CD19 surface proteins and data acquired using BD LSRII running with FACSDiva Software (BD Biosciences). Purity of sorted cells was typically above 95%.

RNA and DNA isolation

Total ribonucleic acid (RNA) was extracted using the RNeasy® Micro kit (Qiagen, Hilden, Germany) according to manufacturer's protocol. RNA was eluted in RNase free water and stored at -80°C. RNA concentrations were determined using the NanoDrop spectrophotometer.

For simultaneous isolation of total RNA and genomic DNA (and protein) for submission to RNA sequencing and whole exome sequencing of CD5+CD19+ FACS-sorted cell the AllPrep DNA/RNA/Protein Mini Kit was used according to manufacturer's protocol.

Whole exome sequencing (WES)

DNA quality was verified using the Genomic DNA ScreenTape assay at a TapeStation System, which was kindly conducted by the Sample Processing Laboratory (SLP) at DKFZ. Samples were submitted to the Genomics and Proteomics Core Facility at DKFZ. Libraries were prepared using the Agilent SureSelect XT Mouse AllExon kit and the library was sequenced in paired end mode on Illumina HiSeq 4000 machine with a read length of 100 base pairs according to the manufacturer's instructions at the DKFZ Genomics and Proteomics Core Facility with three samples per lane.

Mapping and SNV calling of whole exome sequencing data

Raw sequencing reads from whole exome sequencing were aligned with bwa and variant calling was performed using Mutect2 implemented in the nf-core pipeline sarek v3.0.1[4]. Alignment was

performed against GRCm38. The sequenced tails from the TCL-1 donor mice and early time points were used as a panel of normal for the variant calling and dbSNP142 was used for variant filtering. Variants were additionally filtered for at least 3 supporting reads. Due to the missing matching germlines and germline variability between different mice only SNVs were finally analysed. Variant effects were predicted with VEP (GRCm38, version 102) and snpeff (version 5.1, database GRCm38.99). SNVs were annotated with a list of cancer drivers derived from the literature[5].

Analysis of V(D)J rearrangement of BCR of whole exome sequencing data

Bam files were converted into fastq format and used as input for standalone IgBLAST for identifying potential V(D)J rearrangements. The number of unique V(D)J rearrangements was then counted for each sample.

RNA sequencing data processing and functional analysis

RNA was subjected to a quality check using an RNA Nano chip assay on an Agilent 2100 Bioanalyzer. Processing of raw data was conducted by the Genomics and Proteomics Core Facility, DKFZ. Library preparation was performed according to the Illumina TruSeq Stranded protocol on a HiSeq 2000 v4 sequencer, paired-end 125 base pairs with five samples per lane.

Raw sequencing reads from RNA sequencing were aligned to the mouse mm10 reference genome using STAR aligner (v2.5.3a)[6]. Read counting was performed using featureCounts[7]. Gene expression matrix with raw read counts was used as input for DESeq2 R package[8] for downstream analysis like normalization, unsupervised hierarchical clustering with 1000 most variable transcripts and identifying differentially expressed transcripts at a cut off of adjusted P value of less than 0.05. Functional enrichment including gene ontology (GO) analysis was performed on differentially expressed genes as defined by an adjusted P value cutoff of less than 0.05. R (rstudio.com) function prcomp was used for the principal components analysis (PCA) to

examine the gene expression patterns among multiple groups. For gene ontology (GO) analysis differentially expressed genes (DEGs) with a cutoff of less than 0.05 were used.

Sample preparation for mass spectrometry analysis

Either mouse or human CLL cell pellets were resuspended in 150µL of 0.1% RapiGest SF Protein Digestion Surfactant (Waters Corporation) in 100mM ammonium bicarbonate (AmBic) in H2O containing 10mM chloroacetamide (CAA, Sigma) and 40mM tris(2-carboxyethyl)phosphine (TCEP), and sonicated for 15 cycles (30"ON/30"OFF) at 4°C with a PicoBioruptor (Diagenode). Upon BCA protein quantification (Thermo Scientific), samples were heated at 90°C for 5min and subjected to tryptic digestion (Promega) for 18h at 37°C. The pH was lowered at around 2 with trifluoroacetic acid (TFA) in H2O and samples were first incubated for 30 min at 37°C, followed by centrifugation at 18000g for 30min at 4°C. Supernatants were transferred in new PCR tubes and buffer was exchanged via SP3 protein clean up protocol[9,10] to 5mM triethylammonium bicarbonate (TEAB). Peptides were then labeled with TMT10plex (Thermo Scientific) according to manufacturer's instructions. In brief, TMT label reagents were added to sample peptides and incubated for 1h at room temperature, followed by incubation with 5% hydroxylamine for 15min to quench the reaction. Labeled peptides were then combined into TMT10plex experiments and dried in speedvac. Samples were resuspended in 100uL trifluoroacetic acid 0.1% before high pH fractionation on an Agilent 1200 Infinity HPLC system with a Gemini C18 column (3 µm, 110 Å, 100 × 1.0 mm, Phenomenex) using a linear 60 min gradient from 0% to 35% (v/v) acetonitrile in 20 mM ammonium formate (pH 10) at a flow rate of 0.1 ml/min. Elution of peptides was detected with a variable wavelength UV detector set to 254 nm. Forty fractions were collected that were subsequently pooled into 12 or 24 fractions.

Mass spectrometry data acquisition

Dried fractions were resuspended in 0.1% trifluoroacetic acid in H2O, loaded on a trap column (PepMap100 C18 Nano-Trap 100µm x 2cm) and separated over a 25cm analytical column (Waters nanoEase BEH, 75 µm x 250 mm, C18, 1.7 µm, 130 Å,) using the Thermo Easy nLC 1200 (Thermo EasynLC 1200, Thermo Fisher Scientific) coupled to a nanospray source. Solvent A was water with 0.1% formic acid and solvent B was 80% acetonitrile, 0.1% formic acid. During the elution step, the percentage of solvent B increased in a linear fashion from 3% to 8% in 13 minutes, then increased to 16% in 21 minutes, to 50% in 119 minutes, to 95% in 10 minutes where it stayed for 8min followed by decrease down to 3% for the last 9 minutes. Peptides were analyzed on a Tri-Hybrid Orbitrap Fusion or Lumos mass spectrometer (Thermo Fisher Scientific) operated in positive data-dependent acquisition mode with HCD fragmentation. The MS1 and MS2 scans were both acquired in the Orbitrap, with a total cycle time of 3sec. MS1 detection occurred at 60000 resolution, AGC target 1E6, maximal injection time 50 ms and a scan range of 375-1500 m/z. Peptides with charge states 2 to 4 were selected for fragmentation with an exclusion duration of 60s. MS2 occurred with CE 30%, detection in topN mode, and first mass at 110m/z. AGC target was 2E4 and maximal injection time allowed of 94 ms.

Mass spectrometry data processing analysis and visualization

RAW data were processed with Maxquant software (2.0.3.0) including the Andromeda search engine[11,12] or ProteomeDiscoverer v2.1 (ThermoFisher) with Percolator. Peptide identification was performed using either Mouse or Homo sapiens Uniprot database concatenated to a database containing protein sequences of contaminants (canonical and isoform). Default parameters of Maxquant were used with the following modifications: digestion by Trypsin/P and LysC, default variable modification (methionine oxidation and N-terminal acetylation), cytosine carbamidomethylation as a fixed modification. The Instrument set Orbitrap (with precursor tolerance 20ppm, MS tolerance 0.5Da). FDR was set to 1% at both protein and peptide levels.

Match between runs option was enabled, Label-Free Quantification (LFQ), and iBAQ calculated. For further protein analysis, Perseus free software was used[13]. Potential contaminants, reverse proteins, and proteins only identified by sites were removed and only proteins identified with at least one unique peptide in the biological replicates were considered for further analysis. Intensity values were normalized to account for sample mixing error and linear modeling-based batch effect correction r package was employed in the case of comparison between multiple TMT experiments to account for batch-induced variation. Two-sided t-test statistics was used for volcano plots generation based on TMT quantitative information of expressed proteins. FDR was 0.05 and S0 constant was 0.1. Line plots were generated via Perseus software, while pathway enrichment analysis was performed using the Metascape resource[14]. GSEA analysis were performed by means of WebGestalt tool with default parameters[15].

CellTiter-Glo luminescent cell viability assay

Dose-response experiments were performed as previously described[16]. Briefly, compounds (bortezomib, carfilzomib, ibrutinib and ixazomib citrate) were distributed in 384-well cell culture microplates at five different concentrations in ten-fold increments ranging from 1 nM - 10000 nM. Peripheral blood mononuclear cells (PBMCs) from CLL patient samples were co-cultured with irradiated (50 Gy/125 Gy/125 Gy, respectively) CD40L+/APRIL+, BAFF+ and APRIL+ 3T3 fibroblasts (ratio 1:1:1) for 24 h prior to initiation of the experiment to mimic the tumor microenvironment and to prevent spontaneous apoptosis. The patient cells were then separated from the adherent fibroblast layer by carefully re-suspending the culturing medium and transferring it to a separate tube. Single-cell suspension (10000 cells/well in 25 μl) was distributed to each well of the 384-well assay plate. The cells were incubated with the compounds at 37°C for 72h. Cell viability was measured using the CellTiter-Glo luminescent assay (Promega, Madison, WI, USA) according to the manufacturer's instructions. Luminescence was recorded

with an EnVision 2102 Multilabel Reader (PerkinElmer, Waltham, MA, USA). The response readout was normalized to a negative (0.1% DMSO) control.

Immunoblotting

Upon negative selection of murine B cells, whole-cell lysates were prepared in modified RIPA lysis buffer (50 mM Tris-HCl, 150 mM NaCl, 2 mM EGTA and EDTA, and 1% TrionX-100, pH 7.5) with Halt protease and phosphatase inhibitors (Thermo Scientific). Proteins were separated by SDS–PAGE (10%), and then transferred onto a polyvinylidene difluoride membrane (Immobilon-FL, EMD Millipore). The membranes were incubated with blocking buffer containing 1% BSA or 1–5% non-fat dry milk in TBST (Tris-buffered saline with 0.1% Tween-20) at room temperature for 60 min, then with K48-linkage specific polyubiquitin primary antibody (Cell Signaling) at 4 °C overnight and ultimately with HRP-conjugated secondary antibody for 1 h at room temperature. Chemiluminescent signal was detected by Pierce ECL substrate (Thermo Scientific, 32106) and bands were quantified by densitometry using ImagJ freeware (NIH). Scanned images of immunoblots were cropped in the final figures for clarity and conciseness.

Functional analysis

Gene ontology (GO) analyses were performed with either WEB-based GEne SeT AnaLysis Toolkit[17] using KEGG or Reactome as Functional databases or with Metascape[14] with default settings.

Statistics and Reproducibility

Data were analyzed using Prism 5.04 GraphPad software. Comparisons of two different sample groups were performed using unpaired t test with Welch approximation to account for unequal variances, unless otherwise indicated. One-way ANOVA analysis followed by Tukey's multiple comparison test or Kruskal-Wallis test were used for multiple group comparisons. Correlation

between two parameters was calculated using Spearman’s rank correlation coefficient. Values of $p < 0.05$ were considered to be statistically significant. All graphs show means ± SEM, unless otherwise indicated. Sample size of animal studies was determined based on expected variance of read-out. No samples or animals were excluded from the analyses. Several independent experiments were performed to verify reproducibility of results. No randomization was used in animal studies. No blinding was used in animal studies.

Supplemental Table with Reagents or Resources

REAGENT or RESOURCE	SOURCE	IDENTIFIER
**Antibodies**		
Anti-human CD5 (clone UCHT2); 1:100 dilution	Biolegend	Cat# 300605; RRID: AB_314091
Anti-human CD19 (clone HIB19); 1:100 dilution	BD Biosciences	Cat# 555415; RRID: AB_398597
Anti-mouse Ki67 (clone SolA15); 1:400 dilution	eBioscience	Cat # 46-5698-82; RRID: AB_11040981
Anti-mouse CD19 (clone eBio1D3); 1:200 dilution	ThermoFisher	Cat# 11-0193-82; RRID: AB_657666, Cat# 12-0193-81; RRID: AB_657661
Anti-mouse CD19 (clone 6D5); 1:200 dilution	Biolegend	Cat# 115553; RRID: AB_2564000
Anti-mouse CD45 (clone 30F-11); 1:200 dilution	Biolegend	Cat# 103107; RRID: AB_312972<br>Cat# 103131; RRID: AB_893344
Anti-mouse CD5 (clone 53-7.3); 1:200 dilution	BD Biosciences	Cat# 11-0051-81; RRID: AB_464907, Cat# MA5-17784; RRID: AB_2539168, Cat# 17-0051-81; RRID: AB_469330 or Cat# 563194; RRID: AB_2738061
K48-linkage Specific Polyubiquitin (clone D9D5); 1:1000 dilution	Cell Signaling	Cat# 8081; RRID:AB_10859893

REAGENT or RESOURCE	SOURCE	IDENTIFIER
Anti-β-ACTIN (clone AC-15); 1:5000 dilution	Sigma	Cat# A5441;RRID:AB_476744
rat IgG1 isotype control antibody (clone HRPN); 1:400 dilution	BioXcell	Cat# BE0088; RRID: AB_1107775
**Critical commercial assays and other reagents**		
EasySep™ Mouse Pan-B Cell Isolation Kit	StemCell Technologies, Inc.	Cat#: 19844
GentleMacs C tubes	Miltenyi Biotec	Cat#:130-093-237
RNeasy® Micro kit	QIAGEN	Cat#: 74004
RNA RNeasy mini kit	QIAGEN	Cat#: 000074104
Allprep DNA/RNA mini kit	QIAGEN	Cat#: 80204
Illumina TruSeq RNA sample preparation kit v2	Illumina	Cat#: RS-122-2001
Ibrutinib/ PCI-32765 (for *in vitro* purposes)	Selleckchem, Munich, Germany	Cat#: S2680
Ibrutinib/ PCI-32765 (for *in vivo* purposes)	Provided by collaboration partner Prof. Stephan Stilgenbauer	N/A
Carfilzomib/ PR-171	Selleckchem	Cat#: S2853
**Deposited data**		
WES	Sequence Read Archive (SRA)	SUB12126572
RNAseq data	Gene Expression Omnibus (GEO)	GSE215414
MS data	ProteomeXchange Consortium	PXD037314
**Experimental models: organisms/strains**		
Mouse: C57BL/6 (WT)	Charles River Laboratories	N/A

REAGENT or RESOURCE	SOURCE	IDENTIFIER
Mouse: *Eμ*-TCL1 (B6-Tg(Igh-V186.2-TCL1A)3Cro)	Bichi et al., 2002	MGI: 3527221
**Software and algorithms**		
Prism 5.04	GraphPad	https://www.graphpad.com/scientific-software/prism/
FlowJo X 10.0.7 software	FlowJo	https://www.flowjo.com/
FACSDiva Software	BD Biosciences	https://www.bdbiosciences.com/en-us/products/software/instrument-software/bd-facsdiva-software#Overview
R	Wickham et al., 2009<br>Zhao et al., 2014	https://www.r-project.org/
Bioanalyzer 2100 Expert	Agilent Technologies, Santa Clara, USA	https://www.agilent.com/en/product/automated-electrophoresis/bioanalyzer-systems/bioanalyzer-software/2100-expert-software-228259
ImageJ 1.47v	Wayne Rasband, National Institutes of Health, USA	https://imagej.nih.gov/ij/download.html
Maxquant software (2.0.3.0) and Perseus free software	Cox et al Nat Biotech 2008<br>Tyanova et al Nat Meth 2016	https://www.maxquant.org/<br>https://maxquant.net/perseus/

Supplementary Figure 1

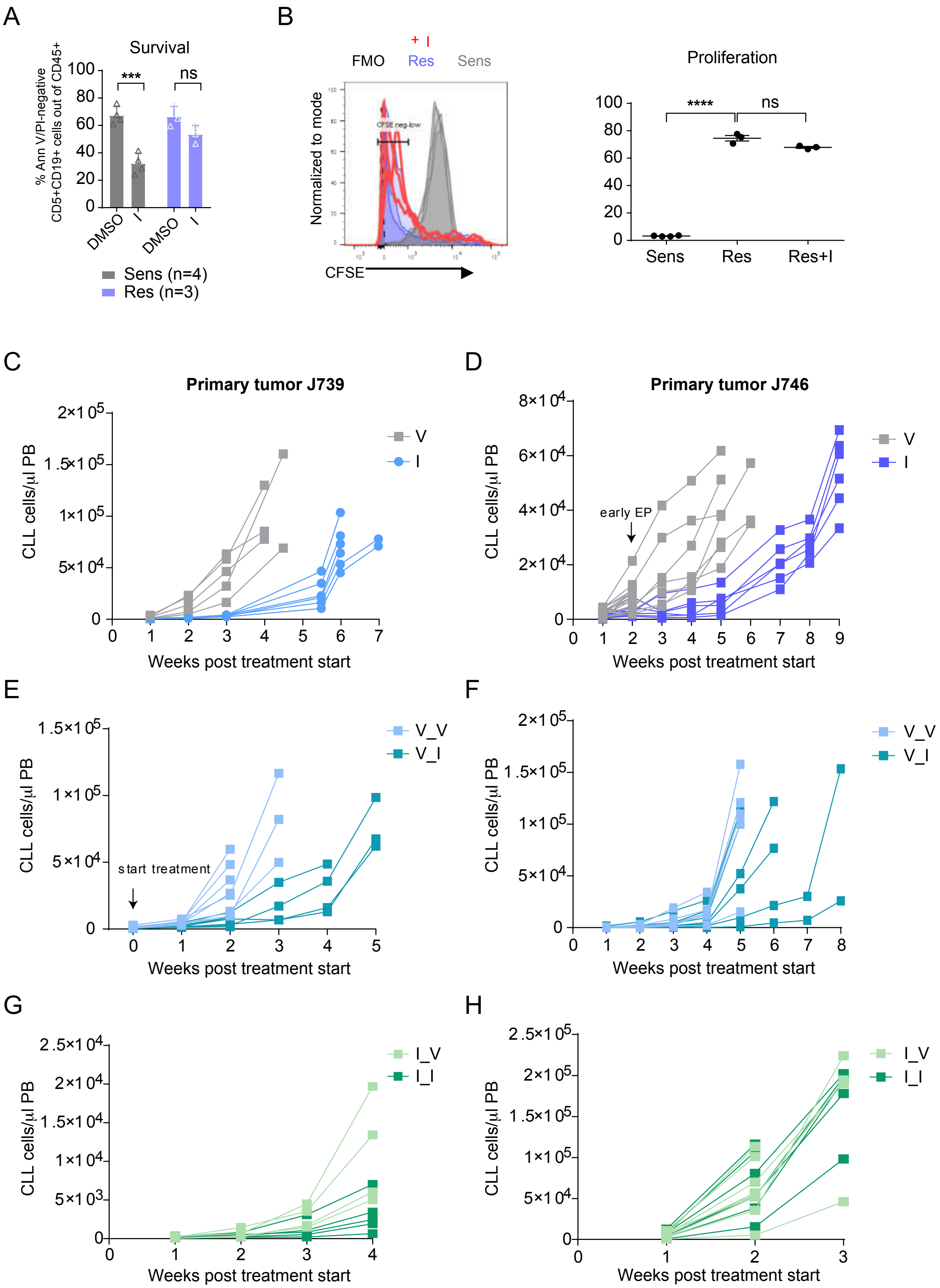

Supplemental Figure 1: Development of ibrutinib-resistance is independent on tumor clone and resistant tumors display increased survival and proliferation potential also *in vitro*. A) Viability of $CD5^+CD19^+$ cells from the spleen was assessed by flow cytometry using Annexin V/7-AAD staining. Sensitive and resistant leukemic cells were CpG-stimulated (0.2µM) and survival was measured after 48 hours. n(Sens)=4; n(Res)=3. Data are mean ± SD and were analyzed by two-tailed unpaired Student's t test, ∗∗∗p=0.0004, ns=0.901. **B)** Flow cytometry histogram (left) and quantification (right) showing proliferation of sensitive (V_week 3) and resistant (I_week 6) leukemic cells. Cells were labeled with CFSE (0.5M) for 10 minutes and incubated in the presence or absence of ibrutinib (1µM) for 72 hours. n(Sens)=4; n(Res)=3. Data are mean ± SD and were analyzed by two-tailed unpaired Student's t test, ∗∗∗∗p<0.0001, ns=0.0687. **C, D)** Absolute numbers of $CD45^+CD5^+CD19^+$ cells in the peripheral blood from mice adoptively transferred with malignant splenocytes isolated from leukemic Eµ-TCL-1 mice from two different tumor clones. Mice were randomized when tumor load was >5000 in the peripheral blood and treatment was started with vehicle or ibrutinib. C: n(V)=6; n(I)=6. D: n(V)=17; n(I)=18. **E-H)** Absolute numbers of $CD45^+CD5^+CD19^+$ cells in peripheral blood of mice injected with vehicle-treated tumors (E, F) or ibrutinib-resistant tumors (G, H) and treated with vehicle or ibrutinib. n≥6. Animals were sacrificed when reaching the moribund status. E: n(V_V)=7; n(V_I)=8. F: n(V_V)=9; n(V_I)=9. G: n(I_V)=7; n(I_I)=8. H: n(I_V)=9; n(I_I)=9.

Supplementary Figure 2

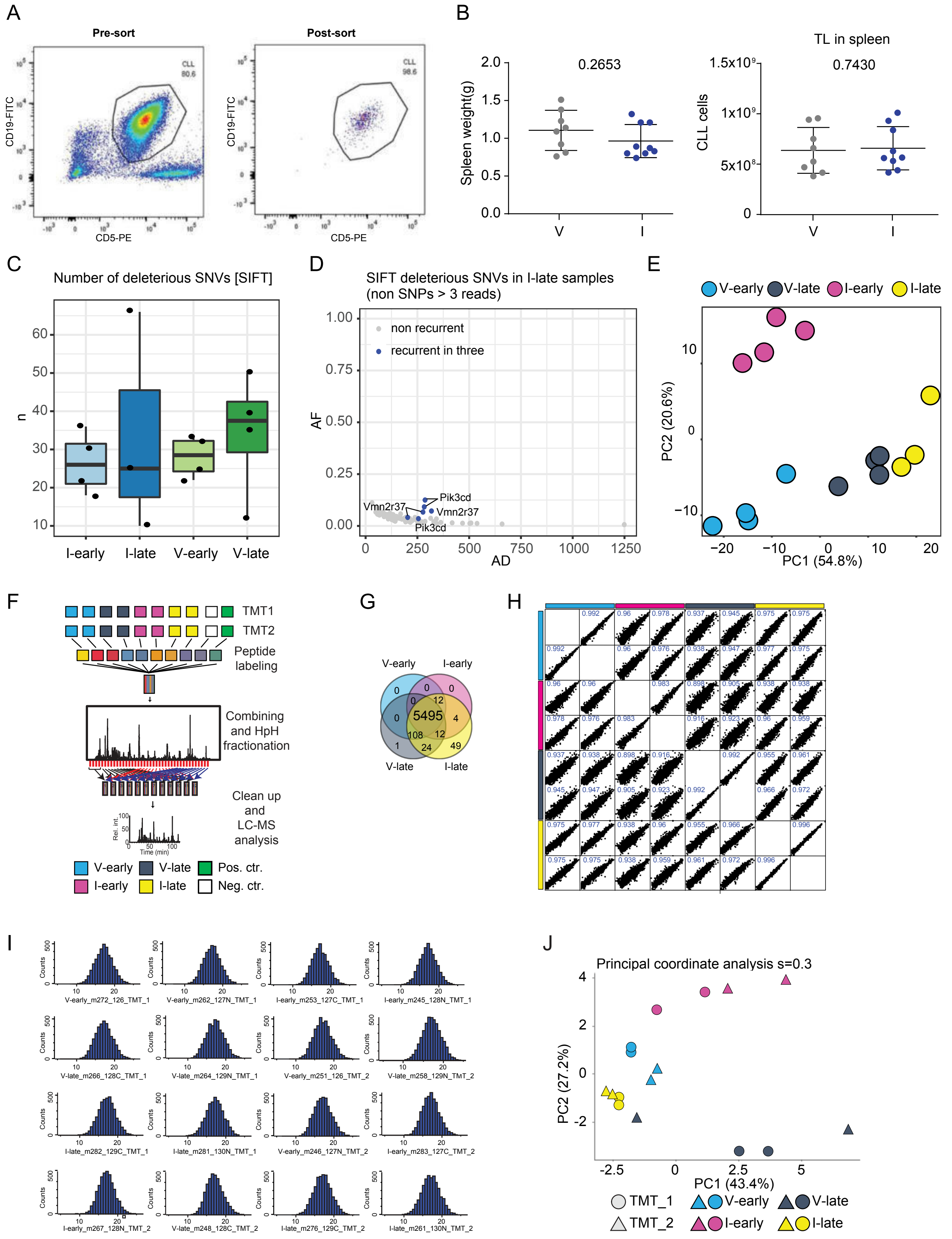

Supplemental Figure 2: The transcriptome of ibrutinib-resistant cells resembles more vehicle-treated rather than ibrutinib-responsive ones. A) Gating strategy and purity assessment of FACS-sorted CLL splenocytes shown in Figure 2. **B)** Spleen weight (left panel) and absolute count of CLL in spleens (right panel) of V-late (V) and I-late (I) samples. Data are mean ± SD and were analyzed by Mann-Whitney test; p values are reported. **C)** Barplot showing the number of deleterious SNVs according to SIFT. Variants with less than three reads support are filtered. Center lines show the medians; box limits indicate the 25th and 75th percentiles; whiskers extend 1.5 times the interquartile range from the 25th and 75th percentiles. **D)** Scatterplot of mutant allele frequency versus read depth of SNVs annotated as deleterious with SIFT. Variants with less than three reads support are filtered and variants overlapping with dbSNP removed. **E)** Principal component analysis (PCA) of transcriptome data obtained by RNA-sequencing of FACS-sorted CLL splenocytes. **F)** Experimental design of TMT-labeled LC-MS/MS of FACS-sorted murine CLL splenocytes. Peptides from listed samples were labeled according to TMT scheme, followed by high-pH (HpH) fractionation before LC-MS/MS analysis. **G)** Venn diagram depicting the number of proteins robustly quantified in each respective group. **H)** Scatter plot showing the correlation of proteins quantified in each sample; Pearson correlation index is reported in blue. **I)** Density distribution for label-free quantification (LFQ) peptide intensities of proteins quantified in the different samples. **J)** PCA for proteomic analysis of mice CLL samples from TMT batch 1 e 2. Ref. corresponds to inter-batches reference samples. Source data are provided as a Source Data file FigS2.

Supplementary Figure 3

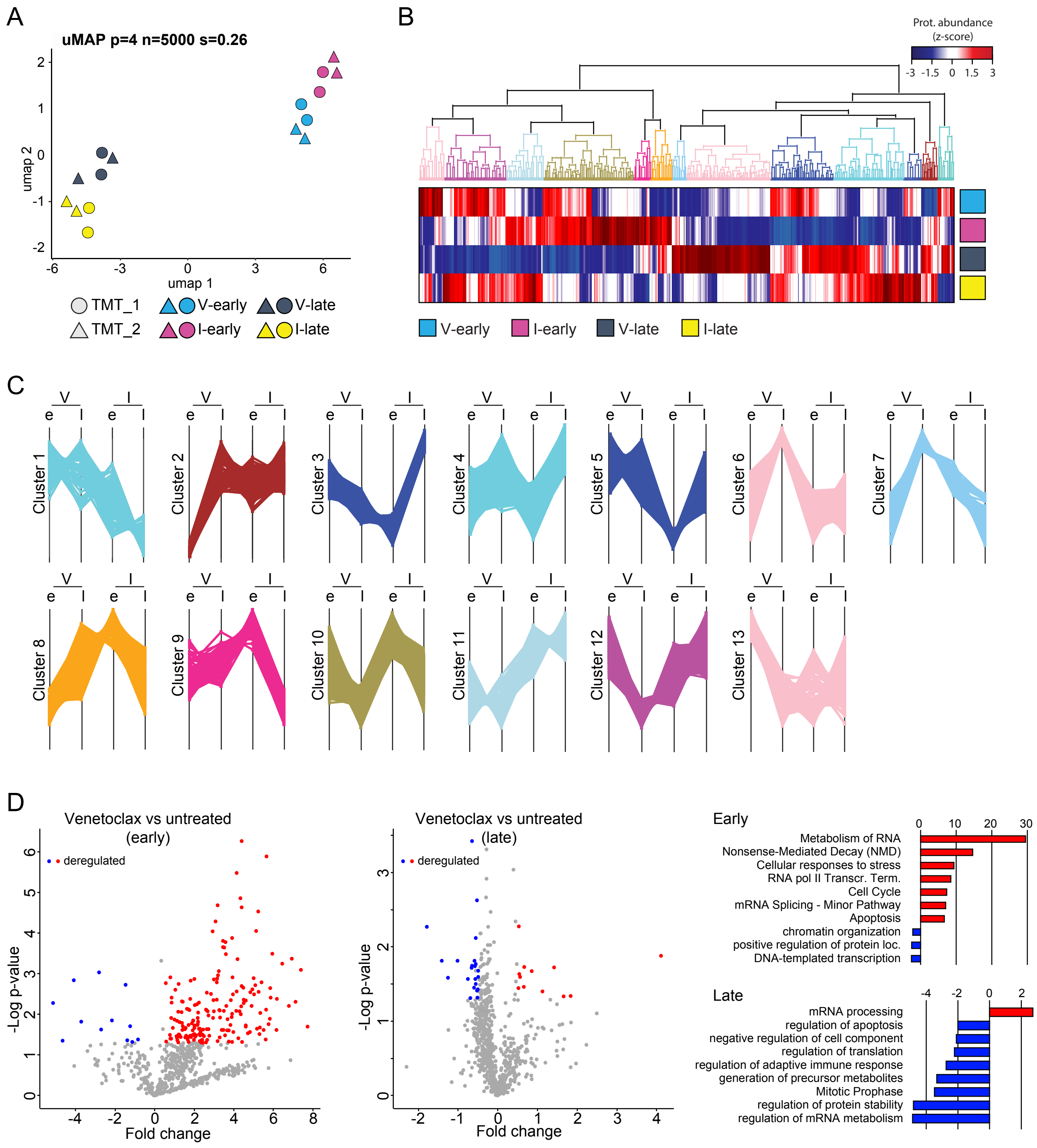

Supplemental Figure 3: The proteome of murine CLL splenocytes is more affected by the stage of disease than by the treatment. A) uMAP plot of proteome data. **B)** and **C)** Heatmap representation (B) and respective protein clusters (C) for significantly deregulated proteins across the four groups of mice analysed (n=4 each group). **D)** Volcano plot representation of proteins dynamically changing upon venetoclax treatment at early (left, n = 2 each group) or late (middle, n = 2 each group) time points, over its respective untreated sample (mean values are depicted). GO terms associated with proteins showing increasing (red) or decreasing (blue) abundance at either early (right-top), or late (right-bottom) time points are shown. Source data are provided as a Source Data file FigS3.

Supplementary Figure 4

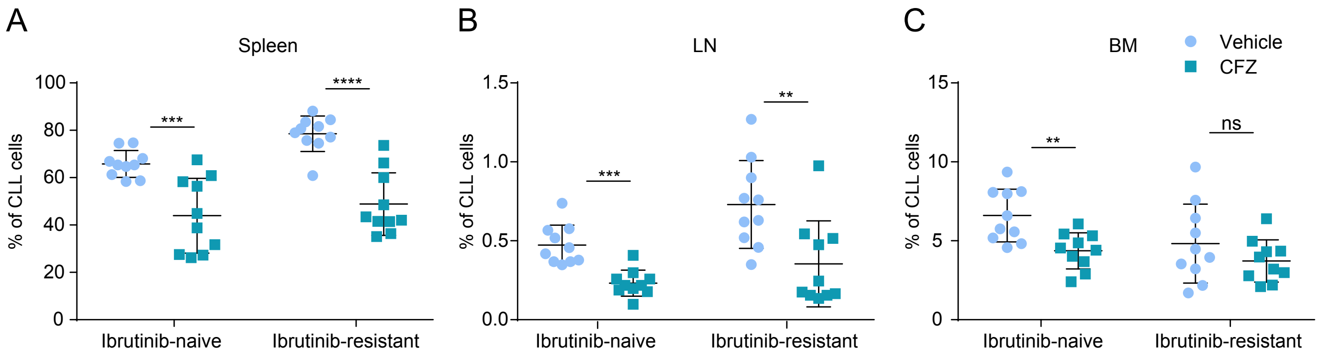

Supplemental Figure 4: Carfilzomib treatment on ibrutinib-resistant and -naïve tumors affects different lymphoid tissues. A-C) Percentage of CLL cells in spleen (A), bone marrows (BM, B), and lymph nodes (LN, C) of mice injected with either ibrutinib-resistant or -naïve tumors and treated with carfilzomib (CFZ, dark blue) or vehicle (light blue). n = 10 each group, in all panels. Data are mean ± SD and were analyzed by two-tailed unpaired Student's t test; A: ***p=0.0010, ****p<0.0001; B: ***p=0.0001, **p=0.0032; C: **p=0.0039, ns=0.3527.

Supplementary Figure 5

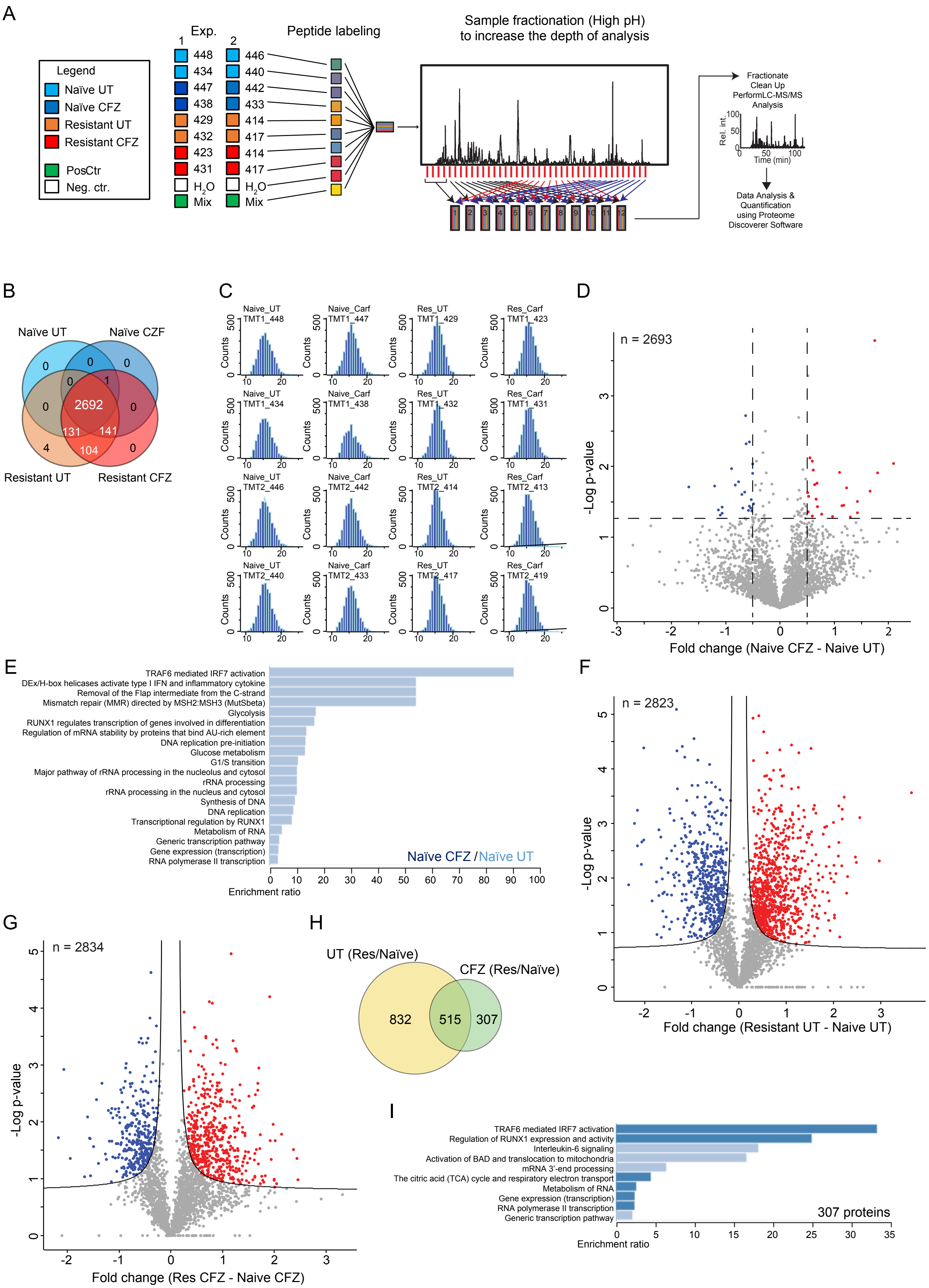

Supplemental Figure 5: Proteome analysis of CFZ-treated tumors reveals a different regulation mechanism in ibrutinib-resistant and ibrutinib-naïve tumors. A) Experimental design of TMT-labeled LC-MS/MS of FACS-sorted murine CLL splenocytes. Peptides from listed samples were labeled according to TMT scheme, followed by high-pH (HpH) fractionation before LC-MS/MS analysis. **B)** Venn diagram depicting the number proteins robustly quantified in each respective group. **C)** Density distribution for label-free quantification (LFQ) peptide intensities of proteins quantified in the different samples. **D)** Volcano plot of proteins significantly deregulated in ibrutinib-naive tumors treated with CFZ (n = 3) or vehicle (n = 3) (two-sided T-test p-value<0.05, fold change >0.5). **E)** Over-representation analysis of significantly deregulated (FDR< 0.05) proteins shown in D with enriched terms. **F, G)** Volcano plots of proteins significantly deregulated in ibrutinib-naïve or ibrutinib-resistant tumors treated with vehicle (F) or CFZ (G); (n = 3 for naïve, n = 4 for resistant, T-test statistics FDR<0.05, S0 = 0.1). **H)** Venn diagram showing the overlap between proteins quantified in vehicle- and CFZ- treated tumors. **I)** Over-representation analysis of the 307 significantly deregulated (FDR< 0.05) proteins specific for carfilzomib resistant (n=4) over carfilzomib Naïve (n=3). Source data are provided as a Source Data file FigS5.

Supplementary Figure 6

A

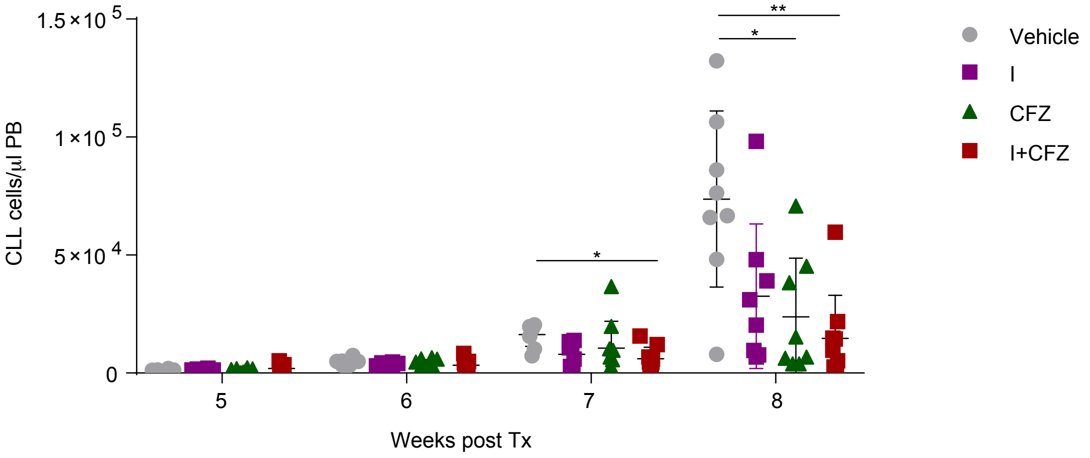

B

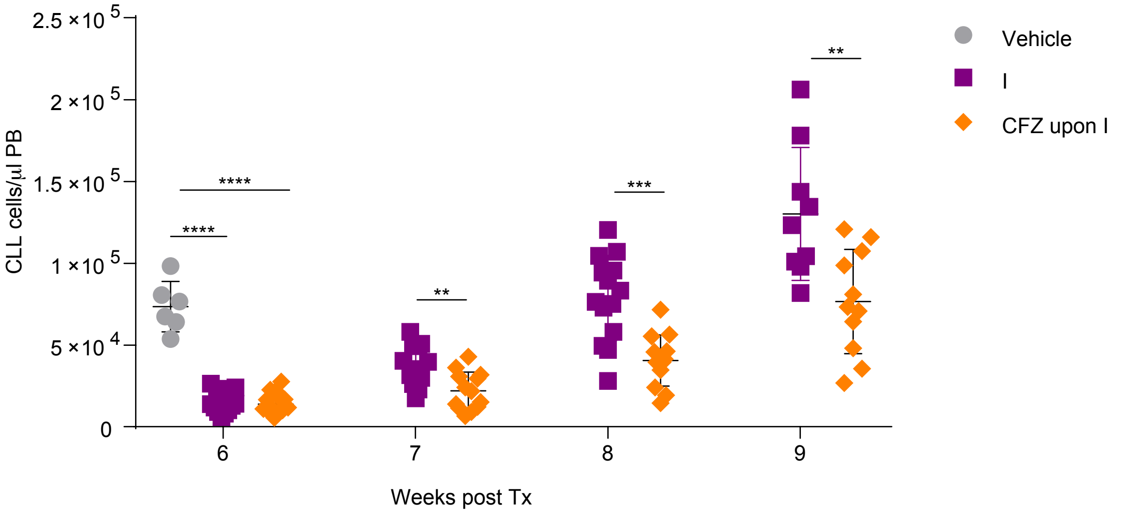

Supplemental Figure 6: Simultaneous ibrutinib and CFZ administration or sequential CFZ treatment upon ibrutinib therapy differently affect tumor progression and survival in mice. A) Absolute numbers of CLL cells in PB from treatment start up to 8 weeks post transplantation (mice from each group still alive). TCL1 mice undergoing ibrutinib (I, n=8, purple square) or carfilzomib (CFZ, n=10, green triangle) monotherapy, the combined therapy (I+CFZ, n=9, red upside-down triangle) or the corresponding vehicle treatment (V, n=8, grey circle) over time. Data are mean ± SD and were analyzed by Kruskal-Wallis test with Dunn´s multiple comparisons test; week 7: ∗p=0.0203; week 8: ∗p=0.0271, ∗∗p=0.00250.01. **B)** Absolute numbers of CLL cells in PB from CFZ treatment start up to 9 weeks post transplantation (mice from each group still alive). TCL1 mice undergoing ibrutinib (I, n=14, purple square), the sequential CFZ administration on mice undergoing ibrutinib therapy (CFZ upon I, n=15, orange rhombus) or the corresponding vehicle treatment (V, n=6, grey circle). Data are mean ± SD and were analyzed by one-way Anova with Tukey´s multiple comparison test (at 6 weeks) or with unpaired Student's t test (at 7, 8 and 9 weeks); week 6: ∗∗∗∗p < 0.0001; week 7: ∗∗p=0.0016; week 8: ∗∗∗p=0.0001; week 9: ∗∗p=0.0039. Only significant comparisons are shown in the figure.

Supplementary Figure 7

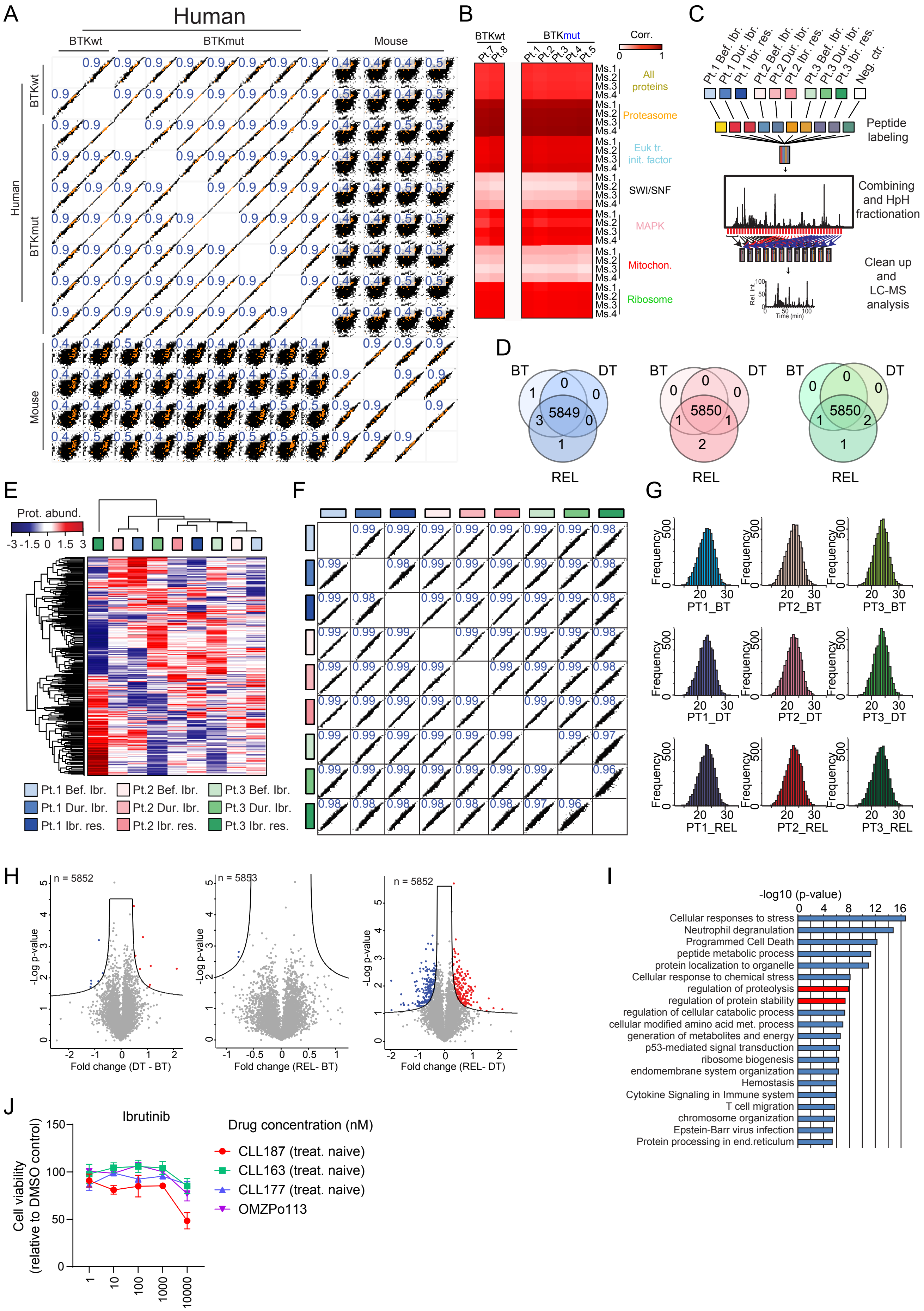

Supplemental Figure 7: Proteome analysis of CLL patients relapsing on ibrutinib. A) Multi-scatterplot showing the Pearson's correlation analysis of proteome data (n=2,840 proteins) from ibrutinib resistant samples of patients with wild type (wt, n=2) or mutant (mut, n=5) BTK, and TCL1-AT mice (n=4). Pearson correlation coefficient as a measure of the strength of the linear relationship of the proteome of two samples each, is reported in blue. Orange dots represent proteasome-related proteins (n=35 proteins). **B)** Heatmap representation of Pearson's correlation coefficients comparing murine ibrutinib resistant samples (n=4; Ms.1-4) and human resistant samples with BTKwt (n=2) or BTKmut (n=5), for all matched proteins or the listed categories of proteins. Number of proteins: All proteins (n=2,840), Proteasome (n=35), Euk tr. init. factor (n=23), SWI/SNF (n=9), MAPK (n=8), Mitochon. (n=24), Ribosome (n=86). **C)** Experimental design of TMT-labeled LC-MS/MS of FACS-sorted human CLL from PBMC. Peptides from listed samples were labeled according to TMT scheme, followed by high-pH (HpH) fractionation before LC-MS/MS analysis. **D)** Venn diagram depicting the number and overlap of proteins robustly quantified in each patient. **E)** Unsupervised hierarchical clustering of all proteins quantified in the three matched samples (n = 5850). **F)** Scatter plot shows the abundance correlation of proteins quantified in the respective sample; Pearson correlation index is reported in blue. **G)** Density distribution for label-free quantification (LFQ) peptide intensities of proteins quantified in each patient sample. **H)** Volcano plots display of proteins significantly deregulated in the different reported comparisons according to two-sided T-test statistics (n = 3 each group) (FDR<0.05, S0 = 0.1). **I)** Barplot shows gene ontologies associated with the 360 proteins significantly deregulated among the different conditions in at least two out of the three patients. Terms linked to proteolysis and protein stability are reported in red. **J)** CellTiter-Glo luminescent cell viability assay performed after 72h exposure to ibrutinib (in triplicate). Graph shows average +/- standard deviation (SD). Experiment was performed on cells from three treatment naive patients (CLL163, CLL177, CLL187) and one ibrutinib-resistant patient (OMZPo113). Source data are provided as a Source Data file FigS7.

Supplementary Figure 8

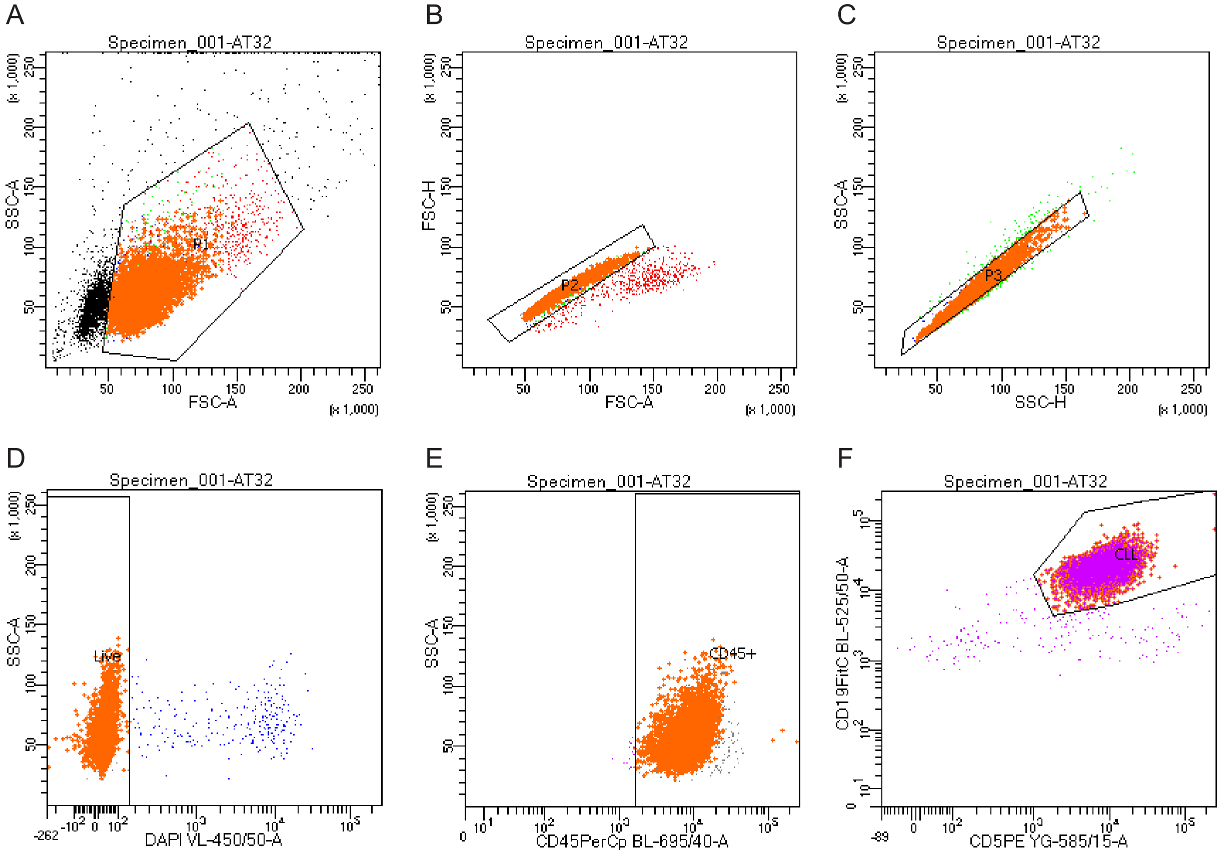

Supplemental Figure 8: FACS gating/sorting strategy. Murine CLL cells shown in Figures 1, 4 and 6 as well as in Supplemental Figures 1, 2, 4 and 6 were isolated according to the following strategy: cells (A)/single cells (B-C)/live cells (D)/CD45+-cells (E)/CD5+-CD19+-cells (F).

Supplementary References

1. Chang, B. Y. *et al.* The Bruton tyrosine kinase inhibitor PCI-32765 ameliorates autoimmune arthritis by inhibition of multiple effector cells. *Arthritis Res Ther* **13** (2011). https://doi.org:ARTN R11510.1186/ar3400
2. Hanna, B. S. *et al.* Depletion of CLL-associated patrolling monocytes and macrophages controls disease development and repairs immune dysfunction in vivo. *Leukemia* **30**, 570-579 (2016). https://doi.org:10.1038/leu.2015.305
3. McClanahan, F. *et al.* PD-L1 checkpoint blockade prevents immune dysfunction and leukemia development in a mouse model of chronic lymphocytic leukemia. *Blood* **126**, 203-211 (2015). https://doi.org:10.1182/blood-2015-01-622936
4. Garcia, M. *et al.* Sarek: A portable workflow for whole-genome sequencing analysis of germline and somatic variants. *F1000Res* **9**, 63 (2020). https://doi.org:10.12688/f1000research.16665.2
5. Wong, J. K. L. *et al.* Association of mutation signature effectuating processes with mutation hotspots in driver genes and non-coding regions. *Nat Commun* **13**, 178 (2022). https://doi.org:10.1038/s41467-021-27792-6
6. Dobin, A. *et al.* STAR: ultrafast universal RNA-seq aligner. *Bioinformatics* **29**, 15-21 (2013). https://doi.org:10.1093/bioinformatics/bts635
7. Liao, Y., Smyth, G. K. & Shi, W. featureCounts: an efficient general purpose program for assigning sequence reads to genomic features. *Bioinformatics* **30**, 923-930 (2014). https://doi.org:10.1093/bioinformatics/btt656
8. Love, M. I., Huber, W. & Anders, S. Moderated estimation of fold change and dispersion for RNA-seq data with DESeq2. *Genome Biol* **15**, 550 (2014). https://doi.org:10.1186/s13059-014-0550-8
9. Hughes, C. S. *et al.* Ultrasensitive proteome analysis using paramagnetic bead technology. *Mol Syst Biol* **10** (2014). https://doi.org:10.15252/msb.20145625
10. Hughes, C. S. *et al.* Single-pot, solid-phase-enhanced sample preparation for proteomics experiments. *Nat Protoc* **14**, 68-+ (2019). https://doi.org:10.1038/s41596-018-0082-x
11. Cox, J. & Mann, M. MaxQuant enables high peptide identification rates, individualized p.p.b.-range mass accuracies and proteome-wide protein quantification. *Nat Biotechnol* **26**, 1367-1372 (2008). https://doi.org:10.1038/nbt.1511
12. Tyanova, S., Temu, T. & Cox, J. The MaxQuant computational platform for mass spectrometry-based shotgun proteomics. *Nat Protoc* **11**, 2301-2319 (2016). https://doi.org:10.1038/nprot.2016.136
13. Tyanova, S. *et al.* The Perseus computational platform for comprehensive analysis of (prote)omics data. *Nat Methods* **13**, 731-740 (2016). https://doi.org:10.1038/Nmeth.3901
14. Zhou, Y. *et al.* Metascape provides a biologist-oriented resource for the analysis of systems-level datasets. *Nat Commun* **10**, 1523 (2019). https://doi.org:10.1038/s41467-019-09234-6
15. Elizarraras, J. M. *et al.* WebGestalt 2024: faster gene set analysis and new support for metabolomics and multi-omics. *Nucleic Acids Res* (2024). https://doi.org:10.1093/nar/gkae456
16. Athanasiadis, P., Ianevski, A., Skånland, S. S. & Aittokallio, T. Computational Pipeline for Rational Drug Combination Screening in Patient-Derived Cells. *Methods Mol Biol* **2449**, 327-348 (2022). https://doi.org:10.1007/978-1-0716-2095-3_14
17. Liao, Y., Wang, J., Jaehnig, E. J., Shi, Z. & Zhang, B. WebGestalt 2019: gene set analysis toolkit with revamped UIs and APIs. *Nucleic Acids Research* **47**, W199-W205 (2019). https://doi.org:10.1093/nar/gkz401
