# Supplementary material for: Longitudinal omics data and preclinical treatment suggest the proteasome inhibitor carfilzomib as therapy for ibrutinib-resistant CLL
Source: Nat Commun. 2025 Jan 26;16:1041. doi: 10.1038/s41467-025-56318-7 (PMC11762753; doi:10.1038/s41467-025-56318-7)
Supplement: Supplementary file 4 — Reporting Summary [file 41467_2025_56318_MOESM4_ESM.pdf]

nature portfolio

Corresponding author(s): Martina Seiffert

Last updated by author(s): Dec 17, 2024

Reporting Summary

Nature Portfolio wishes to improve the reproducibility of the work that we publish. This form provides structure for consistency and transparency in reporting. For further information on Nature Portfolio policies, see our Editorial Policies and the Editorial Policy Checklist.

Statistics

For all statistical analyses, confirm that the following items are present in the figure legend, table legend, main text, or Methods section.

n/a	Confirmed	
☐	☒	The exact sample size (*n*) for each experimental group/condition, given as a discrete number and unit of measurement
☐	☒	A statement on whether measurements were taken from distinct samples or whether the same sample was measured repeatedly
☐	☒	The statistical test(s) used AND whether they are one- or two-sided<br>*Only common tests should be described solely by name; describe more complex techniques in the Methods section.*
☒	☐	A description of all covariates tested
☐	☒	A description of any assumptions or corrections, such as tests of normality and adjustment for multiple comparisons
☐	☒	A full description of the statistical parameters including central tendency (e.g. means) or other basic estimates (e.g. regression coefficient) AND variation (e.g. standard deviation) or associated estimates of uncertainty (e.g. confidence intervals)
☐	☒	For null hypothesis testing, the test statistic (e.g. *F*, *t*, *r*) with confidence intervals, effect sizes, degrees of freedom and *P* value noted<br>*Give P values as exact values whenever suitable.*
☒	☐	For Bayesian analysis, information on the choice of priors and Markov chain Monte Carlo settings
☒	☐	For hierarchical and complex designs, identification of the appropriate level for tests and full reporting of outcomes
☐	☒	Estimates of effect sizes (e.g. Cohen's *d*, Pearson's *r*), indicating how they were calculated

Our web collection on statistics for biologists contains articles on many of the points above.

Software and code

Policy information about availability of computer code

Data collection	FACSDiva (BD Biosciences), Xcalibur™ (ThermoFisher Scientific), OpenLab (Agilent)
Data analysis	FlowJo, ImageJ, MaxQuant, Perseus, GraphPad Prism 5.04, R Studio, Gene Ontology, nf-core pipeline sarek v3.0.1 using Mutect2, Variant effect prediction VEP (GRVm38, version 102), snpeff (GRCm38.99, version 5.1), igBLAST for identification of V(D)J rearrangements, RNAsequencing data aligned with STAR (v2.5.3.a), read counting with featureCounts and analysis with DESeq2 in R. ProteomeDiscoverer v2.1 and WebGestalt for proteoma analyses.

For manuscripts utilizing custom algorithms or software that are central to the research but not yet described in published literature, software must be made available to editors and reviewers. We strongly encourage code deposition in a community repository (e.g. GitHub). See the Nature Portfolio guidelines for submitting code & software for further information.

Data

Policy information about availability of data

All manuscripts must include a data availability statement. This statement should provide the following information, where applicable:

- Accession codes, unique identifiers, or web links for publicly available datasets
- A description of any restrictions on data availability
- For clinical datasets or third party data, please ensure that the statement adheres to our policy

The WES raw data generated in this study have been deposited at BioProject under accession code PRJNA889569 [https://www.ncbi.nlm.nih.gov/sra/PRJNA889569]. The raw RNA sequencing data generated in this study have been deposited in GEO under accession code GSE215414 [https://

www.ncbi.nlm.nih.gov/geo/query/acc.cgi?acc=GSE215414]. The mass spectrometry raw and processed data generated in this study have been deposited to the ProteomeXchange Consortium via the PRIDE (https://www.ebi.ac.uk/pride/)65 partner repository with the dataset identifier PXD037314 and PXD053512 [http://www.ebi.ac.uk/pride/archive/projects/PXD037314, http://www.ebi.ac.uk/pride/archive/projects/PXD053512].
The remaining data are available within the Article, Supplementary Information or Source Data

Research involving human participants, their data, or biological material

Policy information about studies with human participants or human data. See also policy information about sex, gender (identity/presentation), and sexual orientation and race, ethnicity and racism.

Reporting on sex and gender	We provide information on sex for all patients in Table 1. The cohort of samples (n=10) is too small to allow for meaningful conclusions on differences between female and male patients given the high interpatient variability.
Reporting on race, ethnicity, or other socially relevant groupings	Information on race, ethnicity or other socially relevant groupings is not provided as the cohort size was too small to allow for any groupings based on such data.
Population characteristics	Information on genetic aberrations and treatment of patients included in this study is provided in Table 1.
Recruitment	Recruitment of patients was done during their hospital visit and consultation session. Patients were selected based on their clinical and biological characteristics that made them suitable for the research question under study. No compensation was given to the patients.
Ethics oversight	Patient samples were obtained after approval of study protocols by local ethics committees and after obtaining informed consent of patients as indicated in the Methods section of the manuscript. Samples were provided by the B-cell malignancies Biobank at Amsterdam University Centres, Amsterdam, the Netherlands, by the Department of Haematology, Oslo University Hospital, Norway, and by the University Hospital Ulm, Germany. Ethical approval was provided by the Amsterdam University Centres ethical and biobank committee (METC 2013_159), by the Regional Committee for Medical and Health Research Ethics of South-East Norway (2016/ 947), and the Ethics committee of Ulm University, Ulm, Germany (96/08).

Note that full information on the approval of the study protocol must also be provided in the manuscript.

Field-specific reporting

Please select the one below that is the best fit for your research. If you are not sure, read the appropriate sections before making your selection.

[x] Life sciences [ ] Behavioural & social sciences [ ] Ecological, evolutionary & environmental sciences

For a reference copy of the document with all sections, see nature.com/documents/nr-reporting-summary-flat.pdf

Life sciences study design

All studies must disclose on these points even when the disclosure is negative.

Sample size	Sample size of animal studies was determined based on expected variance of read-out.
Data exclusions	No samples or animals were excluded from the analyses.
Replication	Several independent experiments were performed to verify reproducibility of results.
Randomization	No randomization was used in animal studies.
Blinding	No blinding was used in animal studies.

Reporting for specific materials, systems and methods

We require information from authors about some types of materials, experimental systems and methods used in many studies. Here, indicate whether each material, system or method listed is relevant to your study. If you are not sure if a list item applies to your research, read the appropriate section before selecting a response.

Materials & experimental systems

n/a	Involved in the study	
	☒	Antibodies
☒		Eukaryotic cell lines
☒		Palaeontology and archaeology
	☒	Animals and other organisms
	☒	Clinical data
☒		Dual use research of concern
☒		Plants

Methods

n/a	Involved in the study	
☒		ChIP-seq
	☒	Flow cytometry
☒		MRI-based neuroimaging

Antibodies

Antibodies used	See Supplementary Information.<br>Anti-human CD5 (clone UCHT2); 1:100 dilution<br>Anti-human CD19 (clone HIB19); 1:100 dilution<br>Anti-mouse Ki67 (clone SolA15); 1:400 dilution<br>Anti-mouse CD19 (clone eBio1D3); 1:200 dilution<br>Anti-mouse CD19 (clone 6D5); 1:200 dilution<br>Anti-mouse CD45 (clone 30F-11); 1:200 dilution<br>Anti-mouse CD5 (clone 53-7.3); 1:200 dilution<br>K48-linkage Specific Polyubiquitin (clone D9D5); 1:1000 dilution<br>Anti-β-ACTIN (clone AC-15); 1:5000 dilution<br>rat IgG1 isotype control antibody (clone HRPN); 1:400 dilution
Validation	All antibodies were commercially available and validated by the manufacturers, and their specificity was confirmed in several studies that were previously published.

Animals and other research organisms

Policy information about studies involving animals; ARRIVE guidelines recommended for reporting animal research, and Sex and Gender in Research

Laboratory animals	C57BL/6 mice from Charles River;<br>Eμ-TCL1 (B6-Tg(Igh-V186.2-TCL1A)3Cro)
Wild animals	N/A
Reporting on sex	Female mice were used for all experiments as disease development was more homogeneous compared to male mice.
Field-collected samples	N/A
Ethics oversight	All animal experiments were carried out according to institutional and governmental guidelines approved by the local authorities (Regierungspräsidium Karlsruhe, permit numbers G-39/19, G-77/19 and G53/15).

Note that full information on the approval of the study protocol must also be provided in the manuscript.

Clinical data

Policy information about clinical studies
All manuscripts should comply with the ICMJEguidelines for publication of clinical research and a completedCONSORT checklist must be included with all submissions.

Clinical trial registration	N/A
Study protocol	N/A
Data collection	See Table 1 for information on patient samples.
Outcomes	N/A

Flow Cytometry

Plots

Confirm that:

- [x] The axis labels state the marker and fluorochrome used (e.g. CD4-FITC).
- [x] The axis scales are clearly visible. Include numbers along axes only for bottom left plot of group (a 'group' is an analysis of identical markers).
- [ ] All plots are contour plots with outliers or pseudocolor plots.
- [x] A numerical value for number of cells or percentage (with statistics) is provided.

Methodology

Sample preparation	See MM part or Supplementary MM part
Instrument	See MM part or Supplementary MM part (BD LSRII or BD FACSCanto or FACSAria II)
Software	See MM part or Supplementary MM part (FlowJo X 10.0.7 and FACSDiva)
Cell population abundance	See MM part or Supplementary MM part
Gating strategy	See MM part or Supplementary MM part and Suppl. Figure 2A

- [x] Tick this box to confirm that a figure exemplifying the gating strategy is provided in the Supplementary Information.
