# Supplementary material for: Longitudinal omics data and preclinical treatment suggest the proteasome inhibitor carfilzomib as therapy for ibrutinib-resistant CLL
Source: Nat Commun. 2025 Jan 26;16:1041. doi: 10.1038/s41467-025-56318-7 (PMC11762753; doi:10.1038/s41467-025-56318-7)
Supplement: Supplementary file 6 — Source Data [file 41467_2025_56318_MOESM6_ESM.zip › Source_Data/Blot Actin_c-komprimiert-1.pdf]

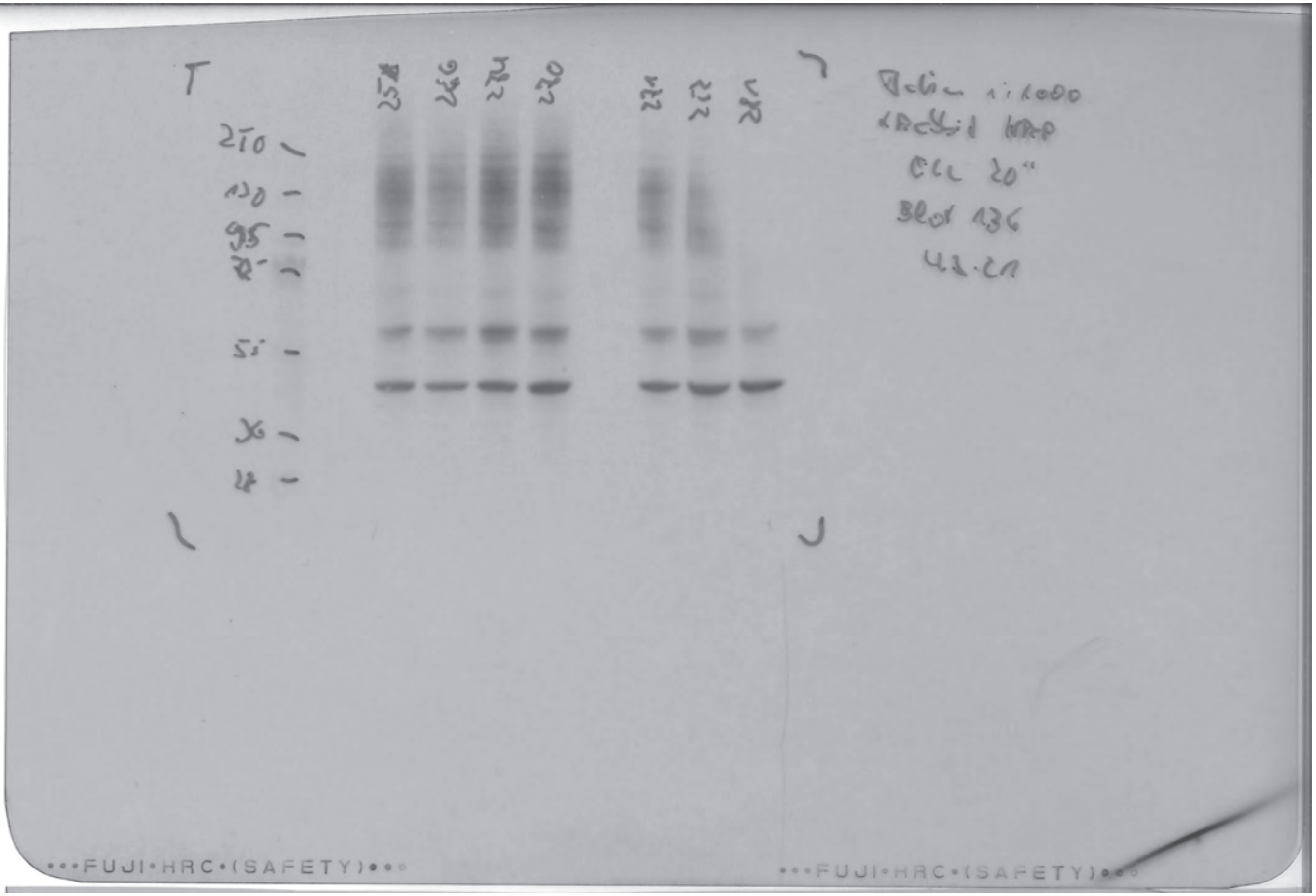
•••FUJI•HRC•(SAFETY)•••
•••FUJI•HRC•(SAFETY)•••
