# Supplementary figures and images for: Longitudinal omics data and preclinical treatment suggest the proteasome inhibitor carfilzomib as therapy for ibrutinib-resistant CLL
Source: Nat Commun. 2025 Jan 26;16:1041. doi: 10.1038/s41467-025-56318-7 (PMC11762753; doi:10.1038/s41467-025-56318-7)

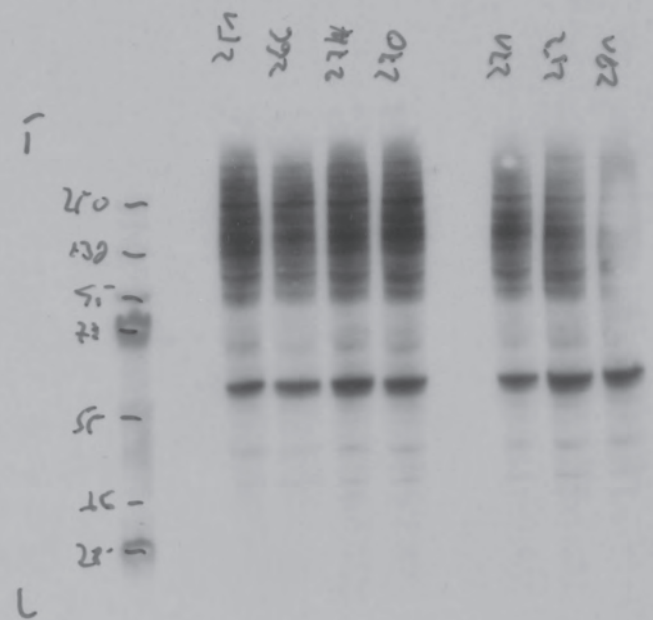

Ubiquitin 1: 1000  
 X-ray 1 HRP  
 ECL 206  
 806 136  
 3.3.21

Supplement: Supplementary file 6 — Source Data [file 41467_2025_56318_MOESM6_ESM.zip › Source_Data/Blot Ubiquitin_c-compressed.pdf]
